# Analysis of outcomes and predictors of long-term survival following resection for retroperitoneal sarcoma

**DOI:** 10.1186/s12893-019-0521-9

**Published:** 2019-06-10

**Authors:** Thomas Malinka, Maxim Nebrig, Fritz Klein, Johann Pratschke, Marcus Bahra, Andreas Andreou

**Affiliations:** Charité – Universitätsmedizin Berlin, corporate member of Freie Universität Berlin, Humboldt-Universität zu Berlin, and Berlin Institute of Health, Department of Surgery, Augustenburger Platz 1, 13353 Berlin, Germany

**Keywords:** Retroperitoneal sarcoma, Predictors, Outcome, Overall survival, Disease-free survival

## Abstract

**Background:**

Retroperitoneal sarcomas (RPS) include a heterogeneous group of rare malignant tumours, and various treatment algorithms are still controversially discussed until today. The present study aimed to examine postoperative and long-term outcomes after resection of primary RPS.

**Patients and methods:**

Clinicopathological data of patients who underwent resection of primary RPS between 2005 and 2015 were assessed, and predictors for overall survival (OS) and disease-free survival (DFS) were identified.

**Results:**

Sixty-one patients underwent resection for primary RPS. Postoperative morbidity and mortality rates were 31 and 3%, respectively. After a median follow-up time of 74 months, 5-year OS and DFS rates were 58 and 34%, respectively. Histologic high grade (5-year OS: G1: 92% vs. G2: 54% vs. G3: 43%, *P* = 0.030) was significantly associated with diminished OS in univariate and multivariate analyses. When assessing DFS, histologic high grade (5-year DFS: G1: 63% vs. G2: 24% vs. G3: 22%, *P* = 0.013), positive surgical resection margins (5-year DFS: R0: 53% vs. R1: 10% vs. R2: 0%, *P* = 0.014), and vascular involvement (5-year DFS: yes: 33% vs no: 39%, *P* = 0.001), were significantly associated with inferior DFS in univariate and multivariate analyses.

**Conclusions:**

High-grade tumours indicated poor OS, while vascular involvement, positive surgical resection margins, and histologic grade are the most important predictors of DFS. Although multimodal treatment strategies are progressively established, surgical resection remains the mainstay in the majority of patients with RPS, even in cases with vascular involvement.

## Background

Soft tissue sarcomas (STS) are a heterogeneous group of rare malignant tumours that can occur in almost any anatomic region [1]. Whereas extremities are reported to be more frequently involved, the incidence of a retroperitoneal origin is expected to be around 0.5 to 1 new cases per 100.000 persons per year, representing approximately about 15% of all STS [2, 3]. Clinical symptoms are often nonspecific and usually a painless, gradually enlarging neoplasm with a median size of 15 to 18 cm is one of the most common findings at diagnosis [4]. The potential to differentiate into many different cell types results in a wide variety of histological entities. The continual development of immunohistochemical and molecular tools requires a continuous reassimilation of classifications, and therefore the substantial comparison is difficult to make [5, 6]. The growth rate of retroperitoneal sarcomas (RPS) varies with the aggressiveness of the tumour. Low-grade tumours may develop over a long period, while high-grade tumours may occur with early symptoms [7].

The retroperitoneum represents a sophisticated anatomical space with multiple vital structures, and therefore RPS is associated with several therapeutic challenges [8]. Especially the close relationship to vital structures may significantly limit the ability to achieve wide resection margins. Currently, various treatment algorithms for patients with RPS have been controversially discussed [9]. Surgical resection involving wide margins, with or without radiotherapy, offers the best chance for a curative intended approach in the absence of metastatic disease [10, 11]. Recent articles highlighted the complexity and technical aspects of resection and strongly advised early referral of these patients to highly specialised centres [12, 13]. Despite recent advances in diagnostic modalities, surgical techniques, and the implementation of more aggressive strategies, RPS is still prone to develop local recurrence approaching up to 50% in some series, even after an apparent complete resection and remains the primary cause of disease-related death [14, 15]. While resection margins status, as well as tumour grading, remain the most important predictors of local recurrence and disease-free survival (DFS), evidence on further prognostic parameters is still limited [8].

The objective of this study was to review our recent experience with RPS and analyse postoperative and long-term oncological outcomes. Besides, we aimed to evaluate factors associated with overall survival (OS) and DFS in this cohort and thus identify patients who may derive the most benefit from a multimodal approach including radical surgery.

## Methods

### Patients’ inclusion criteria

Retrospective single-centre analysis conducted at the Charité – Universitätsmedizin Berlin, Campus Virchow-Klinikum in Berlin, Germany. Following permission from the local institutional review board (EA1/361/14) clinicopathological data of patients who underwent resection for primary RPS between 2005 and 2015 were collected in a prospective database and further reviewed. We excluded recurrent RPS from this analysis, due to divergent management approaches and outcomes. An interdisciplinary tumour board indicated all resections and all patients obtained written informed consent.

### Preoperative assessment

Standard preoperative clinical assessment included physical examination, serum laboratory testing, imaging studies, and an anaesthesia evaluation. Multiphase computed tomography (CT) with contrast agents or magnetic resonance imaging (MRI) were computed to define the dimension and location of the sarcoma and to assess the involvement of adherent structures. A multimodal therapeutic approach was individually formulated and scheduled by a multidisciplinary tumour board, which consisted of surgeons, medical oncologists, specialised radiation therapists, and radiologists, for every patient. Due to the high recurrence rate and aggressive nature of dedifferentiated liposarcoma, neoadjuvant chemotherapy with/without radiation therapy was individually considered for treatment, depending on the individual patient presentation. Therefore, preoperative tissue diagnosis (89%) of retroperitoneal liposarcoma included percutaneous biopsy (image-guided core needle biopsy (CNB) or fine-needle aspiration (FNA)) in order to facilitate an accurate subtype-specific consideration for neoadjuvant therapy.

### Surgical procedure

Perioperative antibiotics [Metronidazole 500 mg (i.v.) and Cefuroxime 1 g intravenously (i.v.)] were routinely given. All procedures were performed in an open surgical approach according to international standards at that time [12]. A midline incision was the most common surgical access facilitating best exposure as well as vascular control. After laparotomy, a complete exploration of the abdominal cavity evaluated local resectability of the sarcoma and the extent of the resection. Especially the need for multivisceral resection was carefully assessed based on local findings such as vascular or other organ infiltration. The surgical procedure was adapted to the anatomic region and intention to achieve radical tumour removal as previously described [12]. Experienced surgeons performed all procedures at the study site.

### Postoperative evaluation

All patients were administered and monitored at a specialised surgical intensive care unit for at least 1 day. The Clavien-Dindo classification was used to grade postoperative complications [16]. Complications within 90 days determined postoperative morbidity. Any in-hospital death following resection defined postoperative mortality. All resected specimens were histologically examined to identify the tumour entity and to evaluate tumour-cell-free surgical margin. Definition of R0 included neither macroscopic nor microscopic tumour cells detectable in postoperative histology. Tumour staging based on the AJCC 8th, Ed. [17]. An interdisciplinary tumour board recommended the use of additional chemotherapy and radiotherapy on a case-by-case basis. Our oncological outpatient clinic assessed long-term follow-up. Besides, a detailed review of medical records, as well as direct communication with the general practitioners tracked patient survival or the documented day of tumour recurrence or death. Fortunately, follow-up data were available for all patients.

### Statistical analysis

Unless otherwise specified, qualitative and quantitative variables are constituted as medians (range) and numbers (frequencies). Postoperative morbidity, mortality, OS, and DFS were defined as primary outcomes. Survival analysis was determined using the Kaplan-Meier method, calculating OS from the date of resection to the date of death or the last follow-up. DFS contained the period from the date of resection to the date of first recurrence or last follow-up. Log-rank tests estimated the significance of univariate analyses. To identify factors associated with survival after resection of RPS, the following clinicopathological characteristics were recorded and analyzed: patient sex, BMI, patient age at resection, tobacco use, tumor entity, histologic grade, staging according to the AJCC 8th Ed., chemotherapy, radiotherapy, surgical resection margin, vascular involvement, ASA physical status, and need for intraoperative transfusion. Furthermore, a Cox multivariate regression model was executed, including all variables associated with survival with *P* < 0.05 in univariate analysis. A *P* value below 0.05 was considered significant, and all statistical analyses were conducted using the SPSS software package, version 24.0 (SPSS, Chicago, IL).

## Results

### Patient characteristics, postoperative morbidity, and mortality

Between 2005 and 2015, 61 patients with primary RPS underwent open resection at our institution. Table 1 summarises clinicopathological data of all patients. The median age was 53 years (12–86), and 48% of patients were male. Median BMI was 25 kg/m^2^ (16–42), and 15 (25%) patients acknowledged consistent tobacco usage. Leiomyosarcoma and dedifferentiated liposarcoma had the highest incidence with 19 (31%) and 14 (23%) patients, respectively. Twelve patients (20%) suffered from well-differentiated liposarcoma, while undifferentiated sarcoma, not otherwise specified, were identified in 13% of all resected tumours. Pleomorphic liposarcoma, malignant peripheral nerve sheath tumours, were detected in 5% of patients, respectively. Histologic grading showed low-grade tumour tissue in 21 (34%) patients, while intermediate grades and high grades were verified in 11 (18%) and 29 (48%) cases, respectively. In terms of the AJCC 8th Ed., the majority of patients (41%) were classified to be Stage IIIB, while Stage IA, Stage IB, Stage II, Stage IIIA and Stage IV were discovered in 3, 28, 7, 13, and 8%, respectively. Forty-nine patients (80%) received no chemotherapy, while ten patients (16%) obtained adjuvant chemotherapy. Two patients (3%) received neoadjuvant chemotherapy. Based on the recommendations of an interdisciplinary tumour board, 44 patients (72%) did not require additional radiotherapy. Four patients (7%) received neoadjuvant radiotherapy, while 13 patients (21%) received adjuvant radiotherapy. In 33 patients (54%) complete tumour removal was achieved. The histological examination discovered persistent tumour cells on the surgical resection margin in 18 patients (30%). In two patients (3%) only an R2 resection could be achieved, while in 8 patients (13%) histological examination did not state sufficient information whether complete tumour resection with surgical margins microscopically negative for tumour cells was successful. Seventeen patients (28%) revealed vascular involvement. In this context, we planned vascular resection in 12 cases already preoperatively. In five cases, preoperative imaging studies underestimated the dimension of RPS and diagnosis of vascular involvement, appeared intraoperatively. The vena cava inferior was the most common vessel involved. In eight cases, segmental resection required a reconstruction by PTFE prosthesis. Within the remaining four cases, direct suture or patch plastic occluded the area of resection. Five patients revealed infiltration of the right renal vein. In all cases, nephrectomy facilitated radical tumour removal. The local recurrence rate of RPS was 41%. ASA physical status included ASA I in 20% of patients, ASA II in 52% of patients, ASA III in 23% of patients, and ASA IV in 5% of patients. In 32 patients (52%) there was no necessity for intraoperative red blood cell concentrate (RBCC) transfusion during the surgical procedure.

**Table 1:** Clinicopathologic characteristics of 61 patients who underwent resection for primary retroperitoneal sarcoma

Characteristics	All Patients (*N* = 61)
Male sex, n (%)	29 (48)
Median age at resection (range), years	53 (12–86)
Median BMI (range)	25 (16–42)
Tobacco use, n (%)	15 (25)
Tumour entity, n (%)	
Leiomyosarcoma	19 (31)
Liposarcoma, dedifferentiated	14 (23)
Liposarcoma, well-differentiated	12 (20)
Undifferentiated sarcoma, NOS	8 (13)
Liposarcoma, pleomorphic	3 (5)
Malignant Peripheral Nerve Sheath Tumors	3 (5)
Other	2 (3)
Histologic grade, n (%)	
Low grade (G1)	21 (34)
Intermediate grade (G2)	11 (18)
High grade (G3)	29 (48)
AJCC 8th Ed., n (%)	
Stage IA	2 (3)
Stage IB	17 (28)
Stage II	4 (7)
Stage IIIA	8 (13)
Stage IIIB	25 (41)
Stage IV	5 (8)
Chemotherapy n (%)	
None	49 (80)
Neoadjuvant	2 (3)
Adjuvant	10 (16)
Radiotherapy	
None	44 (72)
Neoadjuvant	4 (7)
Adjuvant	13 (21)
Surgical resection margin	
R0	33 (54)
R1	18 (30)
R2	2 (3)
RX / Not stated	8 (13)
Vascular involvement, n (%)	17 (28)
Local recurrence, %	25 (41)
ASA physical status, %	
I	12 (20)
II	32 (52)
III	14 (23)
IV	3 (5)
Need for intraoperative transfusions, n (%)	
No red blood cell concentrate	32 (52)
1–2 red blood cell concentrate	13 (21)
≥ 3 red blood cell concentrate	16 (26)
90-day morbidity / Clavien-Dindo ≥3, n (%)	19 (31)
90-day Mortality, n (%)	2 (3)

*BMI* Body-mass-index, *ASA* American Society of Anesthesiologists, *AJCC* American Joint

Committee on Cancer Staging Manual; *NOS* Not otherwise specified

Thirteen patients (21%) required up to two RBCCs during the operation, while 26% of patients demanded more than three RBCCs. Postoperative morbidity, according to the Clavien-Dindo classification ≥3, was 31%, and the 90-day mortality rate was 3%.

### Long-term survival

**Fig. 1:**
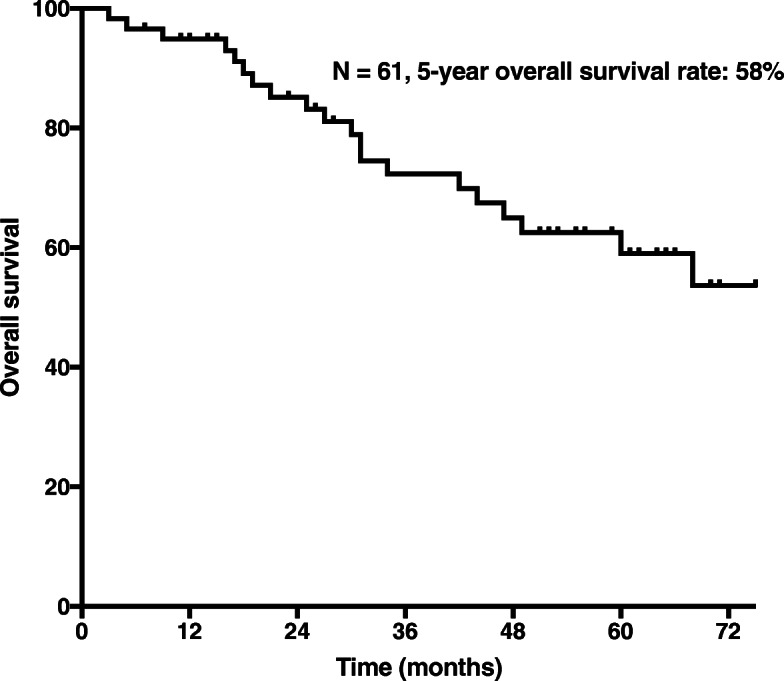
Overall survival in 61 patients who underwent resection for primary RPS

**Fig. 2:**
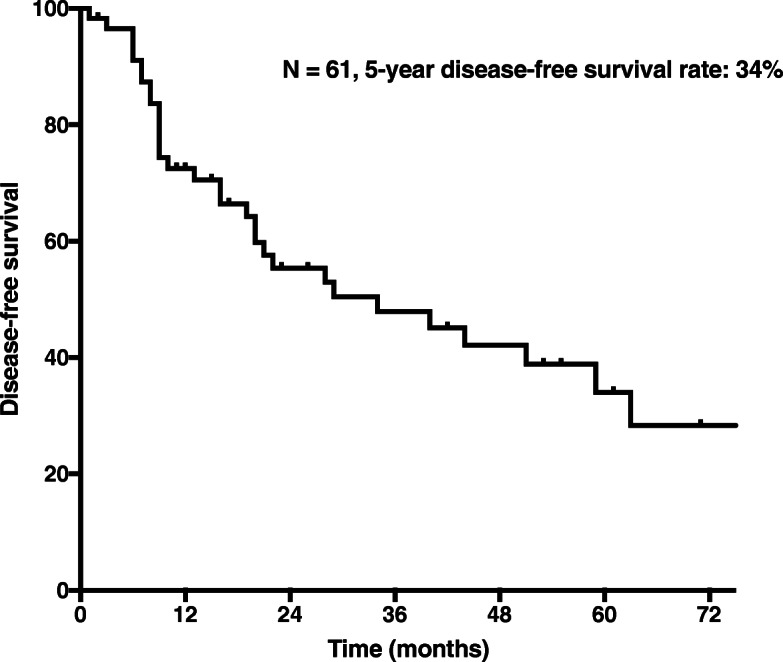
Disease-free survival in 61 patients who underwent resection for primary RPS

After a median follow-up time of 74 months (2–131), the median survival of patients who underwent resection for RPS was 38 months. The 5-year OS rate was 58% (Fig. 1), and 5-year DFS rate was 34% (Fig. 2). The region of resection was the most common site of tumour recurrence.

### Predictors of overall survival

Table 2 summarises the predictors of OS assessed by univariate and multivariate analysis. Factors associated with poor overall survival in the univariate analysis included tumour entity (*P* = 0.018), and histologic tumour grade (*P* = 0.015). In multivariate analysis, only histologic tumor grade (hazard ratio [HR] 2.26, 95% CI 1.08–4.58, *P* = 0.30) independently associated OS (Table 2).

**Table 2:** Univariate and multivariate analysis of clinicopathologic variables associated with overall survival in 61 patients who underwent resection for primary retroperitoneal sarcoma

Variable	N (%)	5-year Overall Survival (%)	Univariate Analysis *P*	Multivariate Analysis*	
				*P*	Hazard Ratio (95% CI)
Sex			.572		
Male	29 (48)	45			
Female	32 (52)	65			
BMI (Body Mass Index)			.483		
< 25	28 (46)	65			
≥25	33 (54)	52			
Patient age at resection			.157		
≥ 60 years	21 (35)	40			
18–59 years	38 (62)	68			
< 18 years	2 (3)	100			
Tobacco use			.522		
Yes	15 (25)	44			
No	46 (75)	60			
Tumour entity			**.018**	NS	
Leiomyosarcoma	19 (31)	71			
Liposarcoma, dedifferentiated	14 (23)	32			
Liposarcoma, well-differentiated	12 (20)	79			
Undifferentiated sarcoma, NOS	8 (13)	31			
Liposarcoma, pleomorphic	3 (5)	39			
Malignant Peripheral Nerve Sheath Tumours	3 (5)	33			
Other	3 (5)	78			
Histologic grade, n (%)			**.015**	**.030**	**2.26 (1.08–4.58)**
Low grade (G1)	21 (34)	92			
Intermediate grade (G2)	11 (18)	54			
High grade (G3)	29 (48)	43			
AJCC 8th Ed., n (%)			.511		
Stage IA	2 (3)	94			
Stage IB	17 (28)	88			
Stage II	4 (7)	68			
Stage IIIA	8 (13)	59			
Stage IIIB	25 (41)	42			
Stage IV	5 (8)	19			
Chemotherapy n (%)			.423		
None	49 (80)	61			
Neoadjuvant	2 (3)	51			
Adjuvant	10 (16)	41			
Radiotherapy			.598		
None	44 (72)	58			
Neoadjuvant	4 (7)	67			
Adjuvant	13 (21)	70			
Surgical resection margin			.186		
R0	33 (54)	61			
R1	18 (30)	45			
R2	2 (3)	0			
RX / Not stated	8 (13)	48			
Vascular involvement			.333		
Yes	17 (28)	36			
No	44 (72)	62			
ASA physical status			.066		
I	12 (20)	90			
II	32 (52)	63			
III	14 (23)	56			
IV	3 (5)	42			
Need for intraoperative transfusions, %			.303		
No RBCC	32 (52)	56			
1–2 RBCC	13 (21)	59			
≥ 3 RBCC	16 (26)	30			

* Cox regression multivariate analysis included all variables with *P* < 0.05 in univariate analysis. *CI* Confidence interval, *NS* Not significant, *BMI* Body-mass-index, *ASA* American Society of Anesthesiologists, *AJCC* American Joint Committee on Cancer Staging Manual, *NOS* Not otherwise specified, *RBCC* Red blood cell concentrate

Entries with a *p*-value of < 0.05 in univariate or multivariate analysis are in boldface. A P value below 0.05 was considered significant.

### Predictors of disease-free survival

**Fig. 3:**
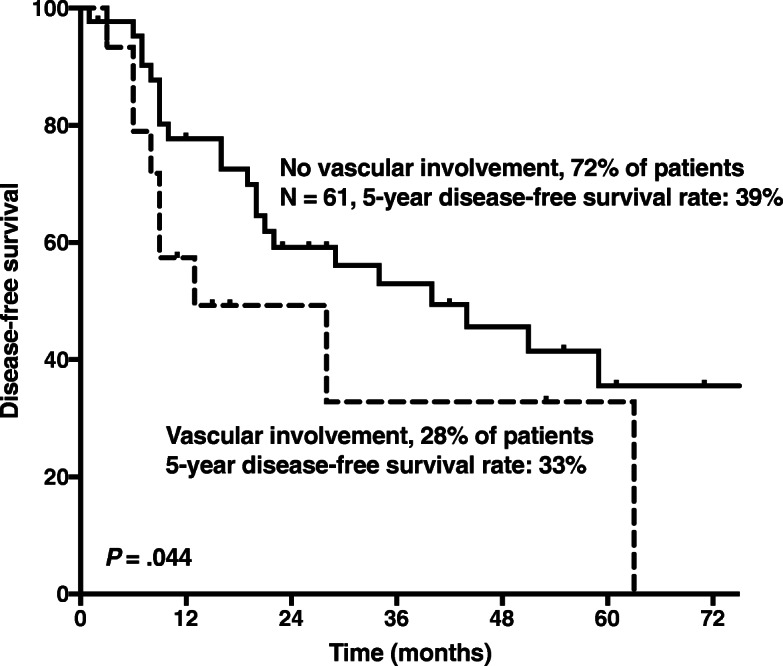
Disease-free Survival by vascular involvement. Patients without vascular involvement had significantly better disease-free survival than those with vascular involvement (*P* = 0.044)

Detailed predictors of DFS, based on the results of univariate and multivariate analysis, are summarized in Table 3. In univariate analysis, factors associated with poor DFS included patient age at resection (*P* = 0.006), histologic tumor grade (*P* = 0.018), classification according to the AJCC 8th Ed. (*P* = 0.004), surgical resection margin (*P* = 0.047) and vascular involvement (*P* = 0.044). In multivariate analysis, histologic grade (HR 2.07, 95% CI 1.17–3.66, *P* = 0.013), positive surgical resection margins (HR 3.12, 95% 1.26–7.72, *P* = 0.014) and vascular involvement ([HR] 5.04, 95% CI 1.90–13.33, *P* = 0.001), were independently associated with DFS (Table 3). In case of vascular involvement (28%), 5-year DFS was 33%, whereas no vascular involvement (72%) resulted in 5-year DFS rate of 39% (*P* = 0.044) (Fig. 3).

**Table 3:** Univariate and multivariate analysis of clinicopathologic variables associated with disease-free survival in 61 patients who underwent resection for primary retroperitoneal sarcoma

Variable	N (%)	5-year Disease-free Survival (%)	Univariate Analysis *P*	Multivariate Analysis*	
				*P*	Hazard Ratio (95% CI)
Sex			.649		
Male	29 (48)	38			
Female	32 (52)	32			
BMI (Body Mass Index)			.635		
< 25	28 (46)	29			
≥25	33 (54)	38			
Patient age at resection			**.006**	NS	
≥ 60 years	21 (35)	16			
18–59 years	38 (62)	47			
< 18 years	2 (3)	50			
Tobacco use			.738		
Yes	15 (25)	40			
No	46 (75)	28			
Tumour entity			.311		
Leiomyosarcoma	19 (31)	31			
Liposarcoma, dedifferentiated	14 (23)	22			
Liposarcoma, well-differentiated	12 (20)	33			
Undifferentiated sarcoma, NOS	8 (13)	25			
Liposarcoma, pleomorphic	3 (5)	39			
Malignant Peripheral Nerve Sheath Tumours	3 (5)	29			
Other	3 (5)	66			
Histologic grade, n (%)			**.018**	**.013**	**2.07 (1.17–3.66)**
Low grade (G1)	21 (34)	63			
Intermediate grade (G2)	11 (18)	24			
High grade (G3)	29 (48)	22			
AJCC 8th Ed., n (%)			**.004**	NS	
Stage IA	2 (3)	85			
Stage IB	17 (28)	71			
Stage II	4 (7)	50			
Stage IIIA	8 (13)	36			
Stage IIIB	25 (41)	28			
Stage IV	5 (8)	0			
Chemotherapy n (%)			.150		
None	49 (80)	34			
Neoadjuvant	2 (3)	50			
Adjuvant	10 (16)	31			
Radiotherapy			.330		
None	44 (72)	33			
Neoadjuvant	4 (7)	50			
Adjuvant	13 (21)	66			
Surgical resection margin			**.047**	**.014**	**3.12 (1.26–7.72)**
R0	33 (54)	53			
R1	18 (30)	10			
R2	2 (3)	0			
RX / Not stated	8 (13)	44			
Vascular involvement			.**044**	**.001**	**5.04 (1.90–13.33)**
Yes	17 (28)	33			
No	44 (72)	39			
ASA physical status			.659		
I	12 (20)	48			
II	32 (52)	36			
III	14 (23)	23			
IV	3 (5)	33			
Need for intraoperative transfusions, %			.899		
No RBCC	32 (52)	38			
1–2 RBCC	13 (21)	41			
≥ 3 RBCC	16 (26)	37			

* Cox regression multivariate analysis included all variables with *P* < 0.05 in univariate analysis. *CI* Confidence interval, *NS* Not significant, *BMI* Body-mass-index, *ASA* American Society of Anesthesiologists, *AJCC* American Joint Committee on Cancer Staging Manual, *NOS* Not otherwise specified, *RBCC* Red blood cell concentrate

Entries with a *p*-value of < 0.05 in univariate or multivariate analysis are in boldface. A P value below 0.05 was considered significant.

## Discussion

This single-centre study examined the postoperative and long-term results of patients treated with radical resection for primary RPS. In this study, 5-year OS and DFS rates of 58 and 34%, respectively, were achieved, which are following results from a previous analysis [4, 18, 19]. Previous studies have aimed to identify predictors for improved OS in patients treated for RPS [20]. Factors related to the primary tumour such as tumour grade and complete resection have been previously described to be the most important predictors of local recurrence and DFS [8].

On the other hand, further controversial studies did not find any survival benefit related to factors such as histological grade, vascular involvement, or tumour size [21, 22]. The convincing benefit of radiotherapy and chemotherapy has been a particular subject of current debate and is still under evaluation [23]. Hence, given data on prognostic factors for patients with RPS are heterogeneous, and we seek to define additional evidence associated with survival in patients with RPS.

In a previous study on this subject, Nathan et al. assessed 1365 patients within the framework of a Surveillance, Epidemiology, and End Results (SEER) program of patients undergoing resection for primary RPS from 1988 to 2005 and reported similar patient characteristics to those reported in our study [22]. Giuliano et al. even assessed 2920 patients from a nationwide database and found mainly the same distribution [20]. Liposarcoma and leiomyosarcoma remain the most common histologic tumour entities. Likewise, the distribution of histologic tumour grade in our analysis is similar. Our median age at diagnosis of 53 years is slightly younger than that reported by both authors, which may indicate advancements in diagnostic modalities. We additionally reported a 5-year OS of 58%, which is following the data from Giuliano et al.

Tumour entity and histologic tumour grade of RPS significantly impact on OS in our univariate analysis. However, in multivariate analysis only histologic tumour grade (G1: 92% vs G2: 54% vs G3: 43%, *P* = 0.015) independently influenced OS. Equivalent to previous findings it might reflect the more advanced nature of tumours with high-grade transformation [8, 15, 18, 24]. Meanwhile, a 5-year OS rate of 58% indicates an improvement compared to the 47% 5-year OS rate reported by Nathan et al. for SEER patients with RPS, which may be related to the more aggressive operative treatment in recent decades compared to that in the late 1980s and 1990s [20, 22]. While both analyses from Nathan and Giuliano did not provide DFS, we were able to report a 5-year DFS rate of 34%.

When addressing DFS, the univariate analysis in our study identified patient age at resection, histological grade, staging according to the AJCC 8th Ed., surgical resection margin and vascular involvement as significant predictive factors. In multivariate analysis, higher tumour grading, positive resection margins, and vascular involvement independently influenced disease-free survival. Previous studies demonstrated that complete, margin-free resection is essential for a potentially curative-intended treatment for RPS [25]. Inability to achieve completeness of tumour resection has a significant adverse prognostic impact and correlates with high-risk for disease-related death [26]. However, while complete surgical resection is the most effective modality for the treatment of RPS, the role of R1 or R2 resection remains controversial. A study performed by Shibata et al. determined clinical outcomes in patients with incompletely resected RPS and noted that for patients with unresectable RPS, incomplete surgical resection provided prolongation of survival and successful symptom palliation [27]. A combined series of 78 patients with RPS who did not undergo R0/R1 resection demonstrated similar findings. In this bi-institutional analysis, the median overall survival was approximately 20 months in the R2 resection cohort, versus 10 months in the cohort who received supportive care or biopsy only [28].

Furthermore, Strom and Mahvi performed a meta-analysis on incomplete resection for RPS and found improved survival at 5 years (44%) compared to survival in patients having biopsy only (17%) [29]. It seems reasonable to presume that tumour debulking or palliative resection can potentially postpone the development of a critical tumour mass with subsequent symptoms. In our analysis, two patients (3%) underwent R2 resection (both dedifferentiated liposarcoma) and demonstrated survival of 18 and 25 months, respectively. Although the management of patients with unresectable RPS is involved and the prognosis is generally poor, we believe that unresectable RPS should not preclude operative intervention per se. Consequently, following interdisciplinary discussion in every case, we may recommend palliative resection to alleviate significant symptoms and prolong survival in highly selected patients.

To date, data regarding vascular involvement and oncological outcomes after resection of RPS is still limited. Previous studies established the feasibility and safety of en-bloc vascular resection for RPS and recommended vascular resection and reconstruction to achieve radical tumour removal when needed [30, 31]. However, whether these complex procedures are associated with a significant prolongation of survival is still unknown. Poultsides et al. described vascular resection and reconstruction during RPS resection with a significantly increased perioperative morbidity, while the oncologic outcome appeared equivalent to cases without significant vascular involvement. The main finding was that the need for vascular reconstruction almost doubled the morbidity of these resections but was associated with a comparable oncologic outcome (local recurrence and overall survival). Our multivariate analysis identified tumour entity and high tumour grade as independent predictors of OS [32]. Our analysis partially confirms these findings, demonstrating comparable OS between patients with and without vascular involvement. However, in our study, patients with vascular involvement recurred earlier. The current study is to our knowledge the first one to report on the association between vascular involvement and tumour recurrence, resulting in a 5-year DFS rate of 72% in patients without vascular involvement compared to 28% in those with proven vascular infiltration. Vascular invasion may be the consequence of biological aggressiveness of the tumour and the need for vascular resection and reconstruction has been examined for a variety of abdominal tumours. For example, patients with pancreatic adenocarcinoma who require portal vein resection have similar OS rates compared to patients not requiring portal vein resection [33]. Although vascular infiltration diminishes DFS in patients with RPS, OS is not affected in our analysis. Consequently, resection of RPS with vascular infiltration can be performed in specialised centres and beneficially prolong survival.

Chemotherapy and radiotherapy did not demonstrate a significant impact on survival. While neoadjuvant therapy is an established treatment for a variety of other tumour entities, evidence on preoperative treatment for RPS is still undetermined, and high-quality trials are needed [3, 34]. The limited efficacy of chemotherapy in RPS may be the result of a variety of reasons. The retroperitoneum represents an ample anatomical space giving primary tumours the ability to grow large before diagnosis, which then results in less effective chemotherapy. Adjuvant radiotherapy is less conventional due to gastrointestinal toxicities, and it is at present not recommended as a standard treatment approach. Hence, preoperative radiation therapy is currently the subject of a European randomised phase III study (STRASS trial) [23, 35]. In summary, the rarity of the disease and lack of high quality randomised controlled data highlights the demand for more international collaborations to characterise the role of systemic therapy and radiotherapy in the management of RPS in order to guide clinicians in their preoperative and postoperative decision-making.

The present study is limited by common biases that are mainly due to the retrospective character of this analysis. Furthermore, treatment regimens are still rather heterogeneous. While extensive surgery remains the mainstay of treatment in RPS, conflicting data on the benefit of neoadjuvant and adjuvant therapies exist, and thus interdisciplinary treatment plans are mainly based on individual preference and expertise of the treating institution [36].

## Conclusion

In conclusion, in our analysis of 61 patients, we found that patient characteristics of RPS is similar to those of other extensive studies, with liposarcoma and leiomyosarcoma being the most common histologies. High-grade tumours indicated poor OS, while histologic grade, positive resection margins, and vascular involvement are the most important predictors of DFS. Although preoperative and postoperative radiotherapy and chemotherapy did not significantly affect survival, procedures tailored to the individual needs of our patients are the current advancement of choice. While effective adjuvant treatment regimens are continuously developed, surgical resection, even in cases with vascular involvement should be individually attempted in selected patients with RPS. Furthermore, international collaborations are mandatory in order to enhance the management of RPS and guide clinicians in their daily decision-making.

## Data Availability

The datasets used and analysed during the current study are available from the corresponding author on reasonable request.

## Abbreviations

CT: Computed tomography
DFS: Disease-free survival
HR: Hazard ratio
MRI: Magnetic resonance imaging
OS: Overall survival
RBCC: Red blood cell concentrate
RPS: Retroperitoneal sarcoma
SEER: Surveillance, Epidemiology, and End Results
STS: Soft tissue sarcoma

## References

[1] Ducimetiere F, Lurkin A, Ranchere-Vince D, Decouvelaere AV, Peoc'h M, Istier L, Chalabreysse P, Muller C, Alberti L, Bringuier PP (2011). Incidence of sarcoma histotypes and molecular subtypes in a prospective epidemiological study with central pathology review and molecular testing. PLoS One.

[2] Trans-Atlantic RPSWG (2015). Management of primary retroperitoneal sarcoma (RPS) in the adult: a consensus approach from the Trans-Atlantic RPS working group. Ann Surg Oncol.

[3] Mullinax JE, Zager JS, Gonzalez RJ (2011). Current diagnosis and management of retroperitoneal sarcoma. Cancer Control.

[4] Hassan I, Park SZ, Donohue JH, Nagorney DM, Kay PA, Nasciemento AG, Schleck CD, Ilstrup DM (2004). Operative management of primary retroperitoneal sarcomas: a reappraisal of an institutional experience. Ann Surg.

[5] Jo VY, Fletcher CD (2014). WHO classification of soft tissue tumours: an update based on the 2013 (4th) edition. Pathology.

[6] Jo VY, Doyle LA (2016). Refinements in sarcoma classification in the current 2013 World Health Organization classification of Tumours of soft tissue and bone. Surg Oncol Clin N Am.

[7] Clark MA, Fisher C, Judson I, Thomas JM (2005). Soft-tissue sarcomas in adults. N Engl J Med.

[8] Strauss DC, Hayes AJ, Thomas JM (2011). Retroperitoneal tumours: review of management. Ann R Coll Surg Engl.

[9] Datta J, Ecker BL, Neuwirth MG, Geha RC, Fraker DL, Roses RE, Karakousis GC (2017). Contemporary reappraisal of the efficacy of adjuvant chemotherapy in resected retroperitoneal sarcoma: evidence from a nationwide clinical oncology database and review of the literature. Surg Oncol.

[10] Strauss DC, Hayes AJ, Thway K, Moskovic EC, Fisher C, Thomas JM (2010). Surgical management of primary retroperitoneal sarcoma. Br J Surg.

[11] Bates JE, Dhakal S, Mazloom A, Constine LS (2018). The benefit of adjuvant radiotherapy in high-grade nonmetastatic retroperitoneal soft tissue sarcoma: a SEER analysis. Am J Clin Oncol.

[12] Bonvalot S, Raut CP, Pollock RE, Rutkowski P, Strauss DC, Hayes AJ, Van Coevorden F, Fiore M, Stoeckle E, Hohenberger P (2012). Technical considerations in surgery for retroperitoneal sarcomas: position paper from E-surge, a master class in sarcoma surgery, and EORTC-STBSG. Ann Surg Oncol.

[13] Learn PA, Bach PB (2010). A decade of mortality reductions in major oncologic surgery: the impact of centralization and quality improvement. Med Care.

[14] Anaya DA, Lahat G, Liu J, Xing Y, Cormier JN, Pisters PW, Lev DC, Pollock RE (2009). Multifocality in retroperitoneal sarcoma: a prognostic factor critical to surgical decision-making. Ann Surg.

[15] Lewis JJ, Leung D, Woodruff JM, Brennan MF (1998). Retroperitoneal soft-tissue sarcoma: analysis of 500 patients treated and followed at a single institution. Ann Surg.

[16] Dindo D, Demartines N, Clavien PA (2004). Classification of surgical complications: a new proposal with evaluation in a cohort of 6336 patients and results of a survey. Ann Surg.

[17] Tanaka Kazuhiro, Ozaki Toshifumi (2018). New TNM classification (AJCC eighth edition) of bone and soft tissue sarcomas: JCOG Bone and Soft Tissue Tumor Study Group. Japanese Journal of Clinical Oncology.

[18] Gronchi A, Miceli R, Shurell E, Eilber FC, Eilber FR, Anaya DA, Kattan MW, Honore C, Lev DC, Colombo C (2013). Outcome prediction in primary resected retroperitoneal soft tissue sarcoma: histology-specific overall survival and disease-free survival nomograms built on major sarcoma center data sets. J Clin Oncol.

[19] Gronchi A, Lo Vullo S, Fiore M, Mussi C, Stacchiotti S, Collini P, Lozza L, Pennacchioli E, Mariani L, Casali PG (2009). Aggressive surgical policies in a retrospectively reviewed single-institution case series of retroperitoneal soft tissue sarcoma patients. J Clin Oncol.

[20] Giuliano K, Nagarajan N, Canner JK, Wolfgang CL, Bivalacqua T, Terezakis S, Herman J, Schneider EB, Ahuja N (2016). Predictors of improved survival for patients with retroperitoneal sarcoma. Surgery.

[21] Pawlik TM, Pisters PW, Mikula L, Feig BW, Hunt KK, Cormier JN, Ballo MT, Catton CN, Jones JJ, O'Sullivan B (2006). Long-term results of two prospective trials of preoperative external beam radiotherapy for localized intermediate- or high-grade retroperitoneal soft tissue sarcoma. Ann Surg Oncol.

[22] Nathan H, Raut CP, Thornton K, Herman JM, Ahuja N, Schulick RD, Choti MA, Pawlik TM (2009). Predictors of survival after resection of retroperitoneal sarcoma: a population-based analysis and critical appraisal of the AJCC staging system. Ann Surg.

[23] ClinicalTrials.gov. NCT01344018. Surgery with or without radiation therapy in treating patients with previously untreated nonmetastatic retroperitoneal soft tissue sarcomas (STRASS) [Internet]. Bethesda: US National Institutes of Health; 2016. [updated 2016 Oct 12]. Available from: https://clinicaltrials.gov/ct2/show/NCT01344018.

[24] Toulmonde M, Bonvalot S, Ray-Coquard I, Stoeckle E, Riou O, Isambert N, Bompas E, Penel N, Delcambre-Lair C, Saada E (2014). Retroperitoneal sarcomas: patterns of care in advanced stages, prognostic factors and focus on main histological subtypes: a multicenter analysis of the French sarcoma group. Ann Oncol.

[25] Porter GA, Baxter NN, Pisters PW (2006). Retroperitoneal sarcoma: a population-based analysis of epidemiology, surgery, and radiotherapy. Cancer.

[26] Youssef E, Fontanesi J, Mott M, Kraut M, Lucas D, Mekhael H, Ben-Josef E (2002). Long-term outcome of combined modality therapy in retroperitoneal and deep-trunk soft-tissue sarcoma: analysis of prognostic factors. Int J Radiat Oncol Biol Phys.

[27] Shibata D, Lewis JJ, Leung DH, Brennan MF (2001). Is there a role for incomplete resection in the management of retroperitoneal liposarcomas?. J Am Coll Surg.

[28] Grobmyer SR, Wilson JP, Apel B, Knapik J, Bell WC, Kim T, Bland KI, Copeland EM, Hochwald SN, Heslin MJ (2010). Recurrent retroperitoneal sarcoma: impact of biology and therapy on outcomes. J Am Coll Surg.

[29] Storm FK, Mahvi DM (1991). Diagnosis and management of retroperitoneal soft-tissue sarcoma. Ann Surg.

[30] Fueglistaler P, Gurke L, Stierli P, Obeid T, Koella C, Oertli D, Kettelhack C (2006). Major vascular resection and prosthetic replacement for retroperitoneal tumors. World J Surg.

[31] Schwarzbach MH, Hormann Y, Hinz U, Leowardi C, Bockler D, Mechtersheimer G, Friess H, Buchler MW, Allenberg JR (2006). Clinical results of surgery for retroperitoneal sarcoma with major blood vessel involvement. J Vasc Surg.

[32] Poultsides GA, Tran TB, Zambrano E, Janson L, Mohler DG, Mell MW, Avedian RS, Visser BC, Lee JT, Ganjoo K (2015). Sarcoma resection with and without vascular reconstruction: a matched case-control study. Ann Surg.

[33] Klein F, Berresheim F, Felsenstein M, Malinka T, Pelzer U, Denecke T, Pratschke J, Bahra M (2018). Routine portal vein resection for pancreatic adenocarcinoma shows no benefit in overall survival. Eur J Surg Oncol.

[34] Ballo MT, Zagars GK, Pollock RE, Benjamin RS, Feig BW, Cormier JN, Hunt KK, Patel SR, Trent JC, Beddar S (2007). Retroperitoneal soft tissue sarcoma: an analysis of radiation and surgical treatment. Int J Radiat Oncol Biol Phys.

[35] Tuan J, Vitolo V, Vischioni B, Iannalfi A, Fiore MR, Fossati P, Orecchia R (2014). Radiation therapy for retroperitoneal sarcoma. Radiol Med.

[36] Fairweather M, Gonzalez RJ, Strauss D, Raut CP (2018). Current principles of surgery for retroperitoneal sarcomas. J Surg Oncol.

